# First worldwide multicenter validation of the POLARIS preclinical polarizer across biological models and imaging paradigms

**DOI:** 10.21203/rs.3.rs-9996713/v1

**Published:** 2026-07-05

**Authors:** Vencel Somai, Hadrien Dyvorne, Galen Reed, Christoph Mueller, Meret Cepero Malo, Catriona Rooney, Andrei Chekushkin, Senay Karaali, Zumrud Ahmadova, Martin Gierse, Michael Keim, Jochen Scheuer, Jonas Handwerker, Felix Josten, Saar Szekely, Pascal Ruetten, Luca Nagel, Miriam Kirst, Sandra Suehnel, Martin Grashei, Franz Schilling, Stephen Lai, Qin Wang, Yunyun Chen, James Bankson, David Gomez-Cabeza, Lluis Mangas, Gergo Matajsz, Alba Herrero Gómez, Vicent Ribas, Irene Marco Rius, Renuka Sriram, Jeremy Gordon, Meetu Wadhwa, Xiao Gao, Tamara Vasilkovska, Daniel Vigneron, Andre Wendlinger, Joshua Kaggie, Ferdia Gallagher, Max Bullock, Rafat Chowdhury, Shonit Punwani, Ashley Shaw, James Quirk, Nicholas Vidas-Guscic, Madison Heady, Caroline Guglielmetti, Cornelius von Morze, Mario Chang, Saket Patel, Roberta Pigliapocchi, Thasin Peyear, Kayvan Keshari, Ilai Schwartz, Stephan Knecht, Myriam Chaumeil

**Affiliations:** NVision Quantum Technologies; NVision Quantum Technologies; NVision Quantum Technologies; NVision Quantum Technologies; NVision Quantum Technologies; NVision Quantum Technologies; NVision Quantum Technologies; NVision Quantum Technologies; NVision Quantum Technologies; NVision Quantum Technologies; NVision Quantum Technologies; NVision Quantum Technologies; NVision Quantum Technologies; NVision Quantum Technologies; NVision Quantum Technologies; NVision Quantum Technologies; Technical University of Munich; NVision Quantum Technologies; Technical University of Munich; Technical University of Munich; Technical University of Munich; The University of Texas MD Anderson Cancer Center; The University of Texas MD Anderson Cancer Center; The University of Texas MD Anderson Cancer Center; The University of Texas MD Anderson Cancer Center; Institute for Bioengineering of Catalonia; Institute for Bioengineering of Catalonia; Institute for Bioengineering of Catalonia; Institute for Bioengineering of Catalonia; Institute for Bioengineering of Catalonia; Institute for Bioengineering of Catalonia; University of California, San Francisco; University of California, San Francisco; University of California, San Francisco; University of California, San Francisco; University of California, San Francisco; University of California, San Francisco; University of Cambridge; University of Cambridge; University of Cambridge; University College London; University College London; University College London; Washington University in St. Louis; Washington University in St. Louis; Washington University in St. Louis; Washington University in St. Louis; Washington University in St. Louis; Washington University in St. Louis; Memorial Sloan Kettering Cancer Center; Memorial Sloan Kettering Cancer Center; Memorial Sloan Kettering Cancer Center; Memorial Sloan Kettering Cancer Center; Memorial Sloan Kettering Cancer Center; NVision Quantum Technologies; NVision Quantum Technologies; NVision Quantum Technologies

**Keywords:** Hyperpolarized 13C, hyperpolarizer, parahydrogen-induced polarization via side-arm hydrogenation (PHIP-SAH), metabolic imaging, hyperpolarized 13C Magnetic resonance imaging

## Abstract

Hyperpolarized ^13^C Magnetic Resonance Imaging (HP-MRI) enables real-time, non-invasive assessment of metabolism in diseases including cancer and neurodegeneration. Broader adoption has been limited by the complexity, duration, and lack of standardization of current hyperpolarization methods. This study evaluated POLARIS Preclinical, a parahydrogen-induced polarization (PHIP) hyperpolarizer designed to streamline production of hyperpolarized ^13^C agents. Four POLARIS systems were deployed across eight international research centers to produce hyperpolarized [1-^13^C]pyruvate doses within 90 seconds. In vitro and in vivo imaging was conducted in multiple animal models using MRI systems operating at 1.4, 3, 7, and 9.4 Tesla. Metabolic conversion of pyruvate to lactate and bicarbonate was successfully measured across all sites. POLARIS enabled rapid, reproducible production of hyperpolarized [1-^13^C]pyruvate and demonstrated consistent performance across instruments, institutions, and field strengths. These results support standardized, high-throughput metabolic MRI for multicenter studies and translational research in oncology, neurology, and cardiovascular disease.

## Introduction

Magnetic Resonance Imaging (MRI) is a cornerstone of preclinical research and clinical diagnostics, providing high-resolution, non-invasive images of anatomical structures and tissue properties across a wide range of diseases. Despite its broad utility, conventional MRI techniques have limited sensitivity for directly probing metabolic processes and dynamic biochemical pathways in living systems. This limitation is particularly significant given that metabolic dysfunction is a hallmark of many leading causes of death, including cancer, cardiovascular disease, and neurodegenerative disorders, where alterations in cellular metabolism often precede structural changes^1,2^. Consequently, the ability to non-invasively visualize biochemical activity *in vivo* is critical for the timely detection of disease, improved understanding of underlying pathophysiology, and the development of targeted, personalized therapeutic strategies that can intervene at earlier and potentially more treatable stages^3,4^.

MRI or MR spectroscopy/spectroscopic imaging (MRS/MRSI) of hyperpolarized (HP) ^13^C-labeled compounds fills this gap by allowing real-time, *in vivo* visualization of metabolic pathways, with [1-^13^C]pyruvate being the most widely investigated HP substrate^1,3,5,6^. Over the past two decades, HP MR of [1-^13^C]pyruvate has been utilized in a broad spectrum of preclinical investigations, with early clinical research studies highlighting its strong potential for translation into clinical practice^2,7,8^. To date, the most extensive application of HP MRI has been in oncology, where HP [1-^13^C]pyruvate has been widely used to quantify elevated metabolic fluxes – such as increased pyruvate-to-lactate conversion – and to monitor treatment response through therapy-induced changes in these metabolic pathways^9–15^. Beyond oncology, hyperpolarized MR has been increasingly applied to the study of renal^16,17^, hepatic^18^, and neurological physiology^19,20^, demonstrating its versatility across organ systems. In the kidney, HP MR has been used to probe renal filtration, acid-base balance, perfusion, and metabolic function, providing insights into functional alterations that are not accessible with conventional imaging^21^. In the liver, hyperpolarized metabolic imaging has enabled the non-invasive characterization of hepatic metabolism, including the metabolic differentiation of fatty liver disease phenotypes^22^. Similarly, in the brain, HP MR studies have begun to elucidate regional metabolic activity and dysfunction, offering new opportunities to investigate neurological physiology and disease mechanisms *in vivo*^23–26^. In addition to HP pyruvate, HP MR has been applied using a variety of other probes to interrogate multiple metabolic pathways. These include agents for assessing extracellular pH^21^, amino acid metabolism^27,28^, TCA cycle flux^23^, tissue perfusion^29^, cell necrosis^30^ and redox status^24^. Methodological advances across sites have expanded the scope of HP MR through the development of optimized pulse sequences^31^, 3D Spectroscopic imaging strategies^32,33^, RF coil designs^34,35^ and quantitative reconstruction methods^36^, enabling improved spatial and temporal resolution and more robust metabolic quantification^37–39^. In parallel, HP MR has been extended to engineered and *in vitro* systems, including organ-on-a-chip platforms, providing controlled environments for high-resolution metabolic studies^40^. Multi-center collaborations have further focused on protocol harmonization and consensus recommendations^5^. These efforts support reproducibility and clinical translation, underscoring the maturation of HP MR from a technical innovation toward a broadly applicable metabolic imaging modality across diverse biological systems and disease contexts.

A central barrier to broader adoption has been the complexity of the dominant hyperpolarization technology, dissolution dynamic nuclear polarization (dDNP). dDNP relies on prolonged polarization cycles (typically 90–120 minutes) at cryogenic temperatures on the order of 1K, strong magnetic fields, and liquid helium infrastructure. This approach requires specialized installations, sterile dose preparations and highly trained personnel, making it operationally limited in many clinical settings, and hindering routine use in patient care. During the past 10 years of SPINlab human dDNP availability (since 2016) over 2000 healthy volunteer subjects and patients have been studied at 17 sites worldwide^5^ with most studies confined to small cohorts at specialized centers. Despite these limitations, these investigations have been critical in establishing the feasibility of HP MRI, demonstrating its safety, and highlighting its potential to non-invasively assess metabolism across multiple organs and disease states. The limited patient numbers y, however, emphasize the emerging unmet need for hyperpolarization technologies that are faster, simpler, and more seamlessly integrated into standard clinical workflows.

Recent advances in catalyst design, precursor formulation, and quantum control have positioned parahydrogen-induced polarization via side-arm hydrogenation (PHIP-SAH) as a rapid, cost-effective approach for producing hyperpolarized (HP) ^13^C-labeled compounds, generating doses suitable for preclinical *in vitro* and *in vivo* studies^41^. Historically, however, reproducibility, standardization, and scalability were difficult to assess, as all PHIP-based hyperpolarizers were custom-built prototypes, limiting direct comparisons across sites.

Breakthroughs in automating hydrogenation, spin-order transfer, hydrolysis, and rapid purification have now culminated in POLARIS—a fully automated, preclinical PHIP-SAH hyperpolarization device engineered to streamline and standardize [1-^13^C]pyruvate production. Using a pre-market version of POLARIS, Nagel *et al*. demonstrated^41^ that PHIP-SAH–polarized [1-^13^C]pyruvate delivers *in vivo* image quality and metabolic readouts, including lactate-to-pyruvate ratios, equivalent to those obtained with d-DNP–polarized [1-^13^C]pyruvate.

We now present a multicenter study, within which we evaluated POLARIS Preclinical, the first commercially available preclinical PHIP-based hyperpolarizer, across eight international imaging centers: University of Cambridge (UK) - UoC, Institute for Bioengineering of Catalonia (Spain) - IBEC, Technical University of Munich (Germany) - TUM, The University of Texas MD Anderson Cancer Center (USA) - MDA, Memorial Sloan Kettering Cancer Center (USA) - MSK, University College London (UK) - UCL, University of California San Francisco (USA) - UCSF, and Washington University in St. Louis (USA) - WashU. Using standardized reagents and protocols, all sites successfully and reproducibly produced HP [1-^13^C]pyruvate within sample production times as short as 90 seconds, totaling over 400 injectable doses. Overall, POLARIS Preclinical systems demonstrated a compact and robust design that is compatible with existing MRI infrastructure, reducing the need for complex setups. Their fast polarization, small footprint (< 1 m^2^), and straightforward operation support more consistent and efficient production of hyperpolarized agents, facilitating broader adoption in preclinical imaging studies and potentially enabling future translation to clinical workflows.

## Results

### The POLARIS polarizer rapidly and reproducibility produced hyperpolarized ^13^C-labeled pyruvate across sites

**Figure 1:**
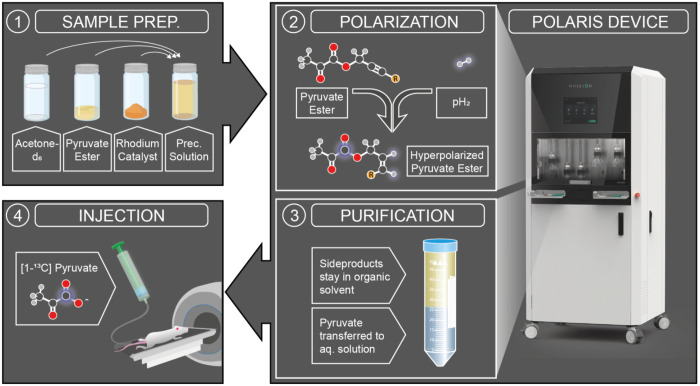
Overview of the POLARIS Preclinical polarizer and its automated hyperpolarization workflow. The system integrates a touchscreen graphical user interface, a reagent area, and internal compartments for parahydrogen and nitrogen gas cylinders and waste collection, with dedicated ports for syringe-based sample loading and extraction. The workflow comprises four sequential steps: (1) sample preparation using a kit with pre-filled vials; (2) sample loading into POLARIS via syringe injection followed by polarization using parahydrogen gas; (3) automated purification to remove catalyst and byproducts; and (4) extraction of the purified hyperpolarized solution for *in vitro* use or intravenous injection *in vivo*.

The POLARIS Preclinical hyperpolarizer (NVision Quantum Technologies GmbH) is a fully integrated system for the rapid production of hyperpolarized ^13^C-labeled contrast agents. The workflow comprises four main steps: (1) sample preparation, (2) polarization, (3) purification, and (4) extraction of the hyperpolarized and purified solution (i.e. dose) ready for *in vitro* or *in vivo* applications. A schematic of POLARIS Preclinical as used for hyperpolarization of pyruvate is shown in Fig. 1. For Step 1, samples were prepared using centrally supplied kits (POLARIS Kit — Pyruvate) that were shipped to all eight participating institutions. Each kit contained sufficient reagents for 20 samples, with each sample consisting of three vials: one with 150 mM vinyl pyruvate ester, another containing HPLC-grade acetone (or acetone-d6 for *in vivo* applications), and a third vial containing 1.5 mM [Rh(dppb)(COD)]BF_6_ catalyst. For each sample, reagents were combined manually immediately prior to polarization (Figure S1). The resulting solution was loaded into the POLARIS Preclinical hyperpolarizer via syringe injection through a luer-lock input port. Steps 2 and 3 are fully automated within each POLARIS unit, incorporating several functional modules to ensure precise and reproducible hyperpolarization. The fluidics and reagent transport system moves the sample and purification reagents between internal components using nitrogen gas, coordinating the sequence of sample transfer and reagent delivery. Parahydrogen gas control regulates the timing and flow of pH_2_ to achieve consistent hydrogenation and polarization transfer. The reactor environment maintains a controlled temperature and pressure, and executes a predefined quantum-control sequence to transfer spin polarization from the hydrogenated side arm to the ^13^C label. Following cleavage of the side-arm, the post-processing module performs phase separation to remove organic residues and residual solvents, directs waste to a sealed container, and delivers the purified hyperpolarized dose to the output port (Step 4). Operation is conducted *via* a touchscreen graphical user interface (GUI) with a stepwise guided protocol, minimizing operator-dependent variability.

**Figure 2:**
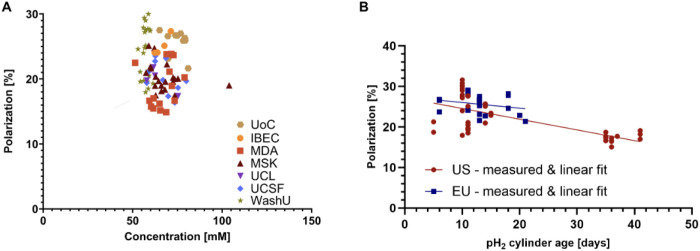
Overview of PHIP-HP [1-^13^C] pyruvate doses **(A)** Dose characterizations (N=91, cylinders filled 5–41 days prior): polarization P= 22.1 ± 4.1 % and concentration C_pyr_= 65.2 ± 2.3 mM **(B)** Polarization fit to pH_2_ cylinder age. Slope = −0.26 %/day (US), −0.14 %/day (EU). Intercept = 27.2% (US), 27.4% (EU).

Across eight sites, over 400 HP [1-^13^C]pyruvate doses were reliably produced in 90 seconds using POLARIS’s standard protocol described above. Parahydrogen was shipped via mail, and pre-filled vials enabled consistent preparation. As shown in Fig. 2A, polarization averaged P = 22.1 ± 4.1% and concentration Cpyr = 65.2 ± 2.3 mM (N = 91, using parahydrogen bottles filled 5–41 days before hyperpolarization). The polarization slope versus cylinder fill day showed minimal drift (Slope = −0.26%/day / −0.14%/day for US- and EU-produced bottles respectively), confirming stability of POLARIS Preclinical over multi-day use (Fig. 2B). The mean acetone concentration in the final injectable solution was 20.5 ± 2.4 mM, corresponding to less than 0.2% of the established LD_50_ in mice. Sample pH was 7.3 ± 0.2.

#### In vitro enzyme kinetics experiments confirmed linear dependence of k_PL_ on LDH activity

**Figure 3:**
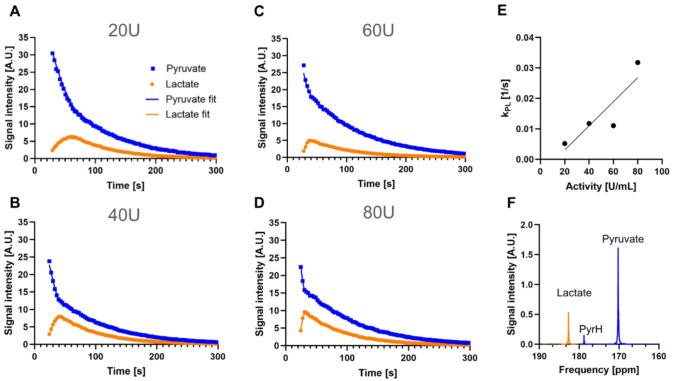
Lactate Dehydrogenase (LDH) HP activity assay. (**A-D**) Pyruvate-to-lactate conversion was measured at LDH activity levels from 20U to 80U in equal steps. Data were fitted to a Michaelis-Menten model. (**E**) Apparent first-order exchange rates (k_PL_ [s^−1^]) were derived from the fit and plotted versus LDH activity. (**F**) Representative ^13^C spectrum shows lactate (183 ppm), pyruvate-hydrate (179 ppm), and pyruvate (171 ppm) peaks with integration ranges for lactate and pyruvate.

Pyruvate-to-lactate conversion as a function of LDH activity was monitored in a benchtop NMR at UCL. *In vitro* enzyme assays exhibited long-lasting pyruvate and lactate signals that were accurately characterized by the fitted Michaelis-Menten model (Fig. 3A–D). The model fitting also confirmed that NADH in the enzymatic solution was in excess, an assumption of central importance in these experiments. Apparent first-order exchange rates k_PL_ derived from the Michaelis-Menten parameters showed linear dependence on LDH activity (R^2^ = 0.77, Fig. 3E). The standard deviation estimated by repeated model fitting due to the stochastic nature of the numerical solver was less than 5%. Figure 3F displays an exemplar spectrum with spectral peak color coding matching those of the time course plots to illustrate ranges of integration.

#### Metabolism of hyperpolarized pyruvate to its downstream products lactate and bicarbonate was detected across model systems, magnetic fields and acquisition protocols

*In vivo* data were collected at five of the eight imaging centers, using a total of 57 mice (BALB/c or C57BL/6, both male and female, 8–14 weeks old, weighing 22–30 g). All imaging experiments were performed using the POLARIS polarizer for hyperpolarization combined with horizontal bore Bruker preclinical MRI systems operating at 3 Tesla (IBEC), 7 Tesla (MDA, TUM), or 9.4 Tesla (UCSF, WashU) field strengths for MR imaging. At WashU additional data was acquired on a 3 Tesla MR Solutions horizontal bore preclinical MRI. The ^13^C signal was detected using either ^13^C surface coils, dual-tuned ^13^C/^1^H volume coils or dedicated ^13^C transmit-receive CryoProbes (Bruker BioSpin).

**Figure 4:**
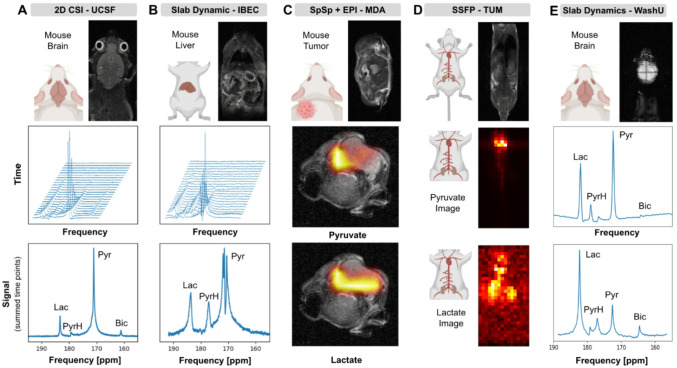
*In vivo*hyperpolarized ^13^C MR data acquired after POLARIS-HP pyruvate injection across sites, organs, field strengths, and acquisition schemes. (**A**) 2D CSI at 9.4 T from healthy mouse brain shows pyruvate metabolism to lactate and bicarbonate. (**B**) Liver (3 T) data show dynamic pyruvate-to-lactate conversion. Splitting of the pyruvate peak is a result of only 0th order shimming being available. (**C**) Thyroid tumor (7 T) showing the expected high lactate production. (**D**) 3D SSFP (7 T) reveals pyruvate in heart and lactate in kidneys, confirming reproducible detection of PHIP-pyruvate metabolism. (E) Sum of spectra showcasing pyruvate metabolism to lactate and bicarbonate in the brain at 3T (top) at 9.4T (bottom).

*In vivo* spectra showed high SNR and detectable signal up to 90 seconds. High lactate production was observed at all field strengths and in all investigated organs including brain, liver and kidney with the thyroid tumors returning the highest lactate signal. Dynamic spectra, in conjunction with anatomical reference scans and schematics outlining the organ of interest, are visualized in Fig. 4A (UCSF − 9.4 T) and Fig. 4B (IBEC − 3 T) in the form of waterfall plots together with the sum of all spectra along the time axis. In Fig. 4C (MDA − 7 T) and Fig. 4D (TUM − 7 T), instead of waterfall plots, 2D pyruvate and lactate maps are shown from the high-resolution EPI and bSSFP scans respectively. Metabolite maps reconstructed from the EPI acquisition captured the high lactate production typical of the thyroid tumors. Pyruvate distribution, reconstructed from the bSSFP scans, provided high-contrast visualization of the cardiac anatomy whereas the highest lactate signal was detected primarily in the kidneys. Figure 4E shows time-summed spectra from brain scans at 3 T and 9.4 T from the same site (WashU). In addition to lactate and pyruvate, bicarbonate signal was consistently detectable for similar duration in the healthy brain in all scans at 9.4 Tesla (Fig. 4A and 4E).

#### Two-pool exchange model revealed elevated k _PL_ values in tumors relative to healthy organs showing close correlation with AUC ratios

**Figure 5:**
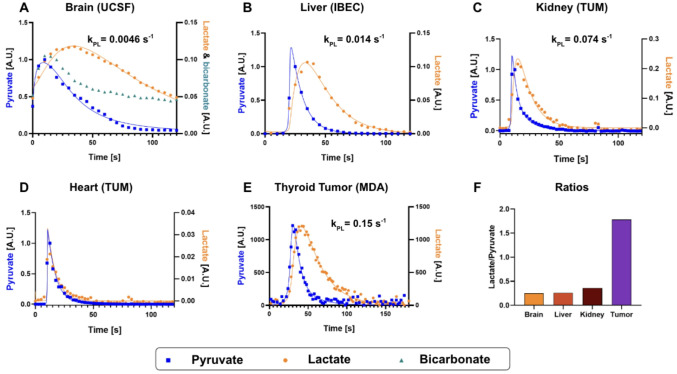
Characterization of metabolic dynamics (A-E) Pyruvate-lactate and Pyruvate-bicarbonate dynamics were extracted from the respective ROI of each in-vivo acquisition. Time courses were fitted with a first-order exchange model with smoothed Heaviside function to account for bolus arrival to extract apparent rate constants (k_PL_). (**F**) Lactate/pyruvate AUC ratios across healthy organs and tumors.

Metabolite time courses of pyruvate and lactate (and bicarbonate where available), extracted from the dynamic spectra, and the fitted two-pool exchange model are shown in Fig. 5. Organ-specific k_PL_ values, estimated from the model fitting, are also displayed in the corresponding subplots except for the heart (Fig. 5D) where no meaningful k_PL_ value could be extracted. Area under the curve (AUC) ratios of lactate and pyruvate are summarized in Fig. 5F with markedly higher lactate production observed in the thyroid tumor relative to the other organs. A high positive correlation of 0.93 was observed between the extracted k_PL_ and AUC ratio values.

## Discussion

Data from four alpha POLARIS Preclinical hyperpolarizers at eight sites yielded over 400 hyperpolarized doses without system failures. Consistent volume, concentration, and polarization confirmed strong reproducibility. Although minor variations in concentration and polarization were observed across different POLARIS devices, reflecting recipe optimization efforts, the values remained tightly clustered around the respective mean especially within data points from the same installation site. Low final acetone concentration in the injectable solution (~ 0.2% of the LD50 for mice^43^) ensured high biocompatibility of the revised recipe for *in vivo* use. The slow decay observed in polarization as a function of parahydrogen cylinder age confirmed a long cylinder shelf life resulting in a large usability window after cylinder shipment and delivery.

In all eight sites, the installation of the POLARIS Preclinical system was notably straightforward, requiring only a single day to complete, which minimizes disruption and enables rapid deployment in laboratory settings. Beyond ease of setup, the system demonstrates strong high-throughput capabilities, allowing for rapid large-scale investigations. This combination of rapid installation and scalability highlights its suitability for large-scale preclinical studies, where time efficiency and consistency across extensive datasets are critical.

*In vitro* enzyme kinetics experiments demonstrated pyruvate-to-lactate conversion that was well described by the fitted simple exchange model. The extracted k_PL_ values closely followed the expected linear dependence on LDH activity^44^, with slight deviations observed at 60 U/mL and 160 U/mL. These discrepancies most likely originated from variance in the mixing of the LDH/NADH-containing buffer solution and the hyperpolarized pyruvate sample that was added to the enzyme solution immediately before the start of the NMR measurement.

A total of 57 *in vivo* experiments were performed at field strengths of 3, 7 and 9.4 Tesla across five sites demonstrated robust detection of pyruvate metabolism to lactate at 3 T, 7 T, and 9.4 T (and to bicarbonate in healthy mouse brain at 9.4 T) as seen on dynamic spectra and images acquired across sites, field strengths and acquisition methods. The acquired spectra featured the expected metabolite peaks and the long lasting signal offered a wide detection window. The reconstructed ^13^C maps in heart, kidney and tumor showed the expected^17,45,46^, markedly different metabolite distribution and dynamics ranging from small amounts of bolus-like lactate immediately following the pyruvate bolus in the heart to large amounts of labelled lactate being produced in the tumor. Estimated k_PL_ values and Lactate/Pyruvate ratios showed good inter-metric consistency as well as organ dependency, in line with literature suggesting that the trace acetone content did not significantly alter the investigated metabolic pathways. Across all sites, no dose-related toxicities were observed in these studies.

In conclusion, POLARIS Preclinical consistently achieved molar polarization levels comparable to DNP with 20-minute inter-dose times at multiple sites worldwide following a simple site-installation that took less than one day. Multiple *in vivo* experiments demonstrated that the POLARIS-produced pyruvate samples are readily compatible with various acquisition techniques such as dynamic slab-selective spectroscopy, slice-selective 2D chemical shift imaging (CSI) or metabolite-selective 3D imaging, as well as different animal models without adverse reactions or altered metabolism. Ease of operation enabled in vivo experiments with as low as two users. The high polarization levels, long T_1_ and the ability to operate POLARIS Preclinical close to NMR or MR systems of various field strengths position POLARIS Preclinical as a flexible and scalable solution for metabolic imaging. By simplifying workflow and reducing dependency on complex infrastructure and large operating team, POLARIS Preclinical opens new avenues for a broader adoption of hyperpolarized metabolic MRI.

## Methods

### Parahydrogen characterization

Parahydrogen-gas was prepared centrally and shipped to each participating site. Hydrogen gas was cooled down to cryogenic temperatures to undergo metal catalyzed ortho-para conversion reaching an enrichment in para state of 99 ± 0.8% during the European preparation process. In the US preparation process the boil-off of liquid hydrogen was collected yielding a para enrichment of 94.5 ± 1.2% while keeping oxygen and other paramagnetic impurities below 1 ppm. The enrichment of the para state was verified by means of Raman spectroscopy. The resulting para state-enriched hydrogen (also referred to as parahydrogen) was filled into 5L aluminium cylinders pressurized to 8–15 bars.

### Hyperpolarized sample characterization

Precursor samples intended for *in vitro* characterization were prepared using HPLC-grade protonated acetone (n = 38) or acetone-d6 as solvent (n = 53). Samples were characterized by measurements of ^13^C polarization (% and T_1_ decay), concentration of hyperpolarized pyruvate (mM), pH, temperature, sample volume and concentration of remaining acetone solvent (mM). The polarization was measured 20 to 30 seconds post-production on a 60 MHz benchtop NMR system (Nanalysis NMReady 60PRO) using inverse-gated decoupling, a 5° excitation and subsequent signal detection over a 200 ppm bandwidth. The same acquisition protocol was repeated 100 times immediately afterward to measure the T_1,13C_ relaxation time constant of the sample and to confirm the complete decay of hyperpolarization. For quantification of enhancement, the first hyperpolarized ^13^C peak integral *S*_13*C,HP*_ was directly compared to a thermal ^13^C reference integral *S*_13*C,REF*_ of labelled methanol (*C*_*REF*_ = 24.66 M; 99% ^13^C atom; flip angle (FA) = 5°; number of averages (NA) = 32; repetition time (TR) = 60 s), acquired under identical conditions:

$$P=\frac{S_{13C,HP}}{S_{13C,REF}}\cdot \frac{C_{REF}}{C_{Pyr}}\cdot \text{tanh}\left(\frac{\hbar \;\gamma_{13C}B_{0}}{2k_{B}T}\right)$$

No T_1_ correction or backprojection of the polarization was applied, i.e. the values stated represent the polarization at time of measurement.

The concentration *C*_*Pyr*_ was quantified from ^1^H spectroscopy (FA = 90°, NA = 4, TR=60s, Bandwidth (BW)=100ppm) of the fully relaxed sample by direct comparison of the ^1^H peak integral *S*_1*H,Pyr*_ of the produced sample and the ^1^H peak integral *S*_1*H,REF*_ of a DMSO in D_2_O standard reference at known concentration (*C*_*REF*_ = 0.492 M), and by taking the different numbers of ^1^H per molecule *N*_1*H*, …_ into account:

$$C_{Pyr}=\frac{S_{1H,Pyr}}{S_{1H,REF}}\cdot \frac{N_{1H,REF}}{N_{1H,Pyr}}\cdot C_{REF}$$

The remaining acetone concentration in the produced pyruvate samples was calculated similarly. Furthermore, before injection, pH was determined using standard pH test strips (Cytiva Whatman, USA; pH 6 - pH 10, pH 0.5 steps).

#### In vitro MR acquisitions

Enzyme solutions were freshly prepared immediately before each measurement by dissolving varying amounts of lactate dehydrogenase (LDH, Sigma Aldrich, 10 mg/mL, 550 U/mg protein) and 0.0258 g nicotinamide adenine dinucleotide (NADH) in 2 mL of phosphate-buffered saline (PBS). LDH volumes ranged from 7.26 to 29.04 μL across four equidistant steps, producing solutions with enzymatic activities of 20, 40, 60 and 80 U/mL. After thorough vortex mixing, 250 μL of each solution was transferred into a standard 5mm NMR tube and allowed to equilibrate to 33°C inside a 60 MHz benchtop NMR system (Nanalysis NMReady 60PRO) at UCL. The temperature of the HP pyruvate sample was measured immediately upon extraction, and 250 μL was added to the enzyme solution, yielding a final NMR sample volume of 500 μL. Time-resolved ^1^D ^13^C spectra were then acquired to monitor pyruvate-tolactate conversion at the different enzyme activity levels. Spectra were recorded using 100 dynamic repetitions with a repetition time (TR) of 2 s, a bandwidth of 3125 Hz, a flip angle of 2°, and 4096 complex points per acquisition.

#### In vivo MR acquisitions

All animal experiments were conducted in accordance with institutional and national ethical guidelines and approved by the respective institutional animal care and use committees (IACUC) or equivalent regulatory bodies at each participating site.

Across sites, all animals were maintained under standard housing conditions with ad libitum access to food and water and a 12-hour light/dark cycle. For all metabolic imaging, mice were anesthetized using isoflurane (1.5–2.0% in oxygen) during tail vein catheterization, injection, and MR acquisitions, during which they were positioned in dedicated animal holders with integrated respiratory monitoring. Core body temperature was maintained at 36–37°C using heated circulating water or warm air during the imaging session. HP [1-^13^C]pyruvate was injected intravenously immediately after production at a volume of 200–350 μL per mouse over 10–12 seconds.

For each site, the following acquisition strategies were employed:

- IBEC - Slab Dynamic Imaging on ParaVision-360.3.4. software version: Used to monitor metabolite kinetics in single-slice applications (e.g., brain or liver), with acquisition initiated within 10 s of injection. One-dimensional ^13^C spectra comprising 2048 complex points were acquired with a 1333 Hz bandwidth and 15° flip angle from an 8 mm-thick slab centered on the liver, using 50 dynamic repetitions and a 5 s inter-scan delay.
- MDA - A custom sequence using a spectral-spatial excitation with interleaved free induction decay (FID) and echo-planar imaging (EPI) readouts, implemented on ParaVision-6.0.1, was used for dynamic metabolic tracking in tumor-bearing animals^42^. Acquisition parameters included TE/TR = 13.99/50.0 ms, 90 dynamic repetitions with an inter-scan delay of 2000 ms. Flip angles were set at 10° (pyruvate) and 30° (lactate). The field of view was 40 × 40 × 10 mm^3^.
- TUM - Fast Imaging with Steady-state Precession (FISP) on ParaVision-7.0.0. software version: 3D whole body scan to monitor the distribution of the injected pyruvate bolus and the subsequent conversion to lactate at a FOV of 56 × 28 × 21 mm^3^ encoded into a 32 × 16 × 12 matrix sampled at 5kHz bandwidth (TE/TR = 3.26/6.52 ms). Metabolite selective excitation pulses with 6° and 40° flip angle were employed to image pyruvate and lactate respectively over 150 × 2 interleaved dynamics with an inter-scan delay of 1.25 s.
- UCSF - Free induction decay chemical shift imaging (FID-CSI) on ParaVision-360.3.5. software version: 3 mm slice excitation with 10° and subsequent acquisition of a center-out, phase-encoded free-induction decay (FID) to achieve localized spectroscopy with a 0.76 × 0.76 mm^2^ to 1.0 × 1.0 mm^2^ in-plane resolution, and bandwidths ranging from 3–5.3 kHz.
- WashU - Slab Dynamic Imaging with spectral-spatial excitation on ParaVision-360.3.5 software version.: enables monitoring metabolite kinetics in single-slice applications while applying different, metabolite specific flip-angles. One-dimensional ^13^C spectra comprising 2048 complex points were acquired with a 4065 Hz bandwidth and 5°, 20° and 90° flip angle on pyruvate, lactate and bicarbonate resonances respectively from an 5 mm-thick slab centered on the brain, using 64 dynamic repetitions and a 3 s inter-scan delay.
- A similar protocol was used on the 3T MR Solutions scanner to record an array of one-dimensional ^13^C spectra (16384 complex points, 64 dynamic repetitions, 3 s inter-scan delay) from an 8 mm slab centered on the brain using slice-selective excitation.

### Data Analysis and Quantification

For sample characterization, ^1^H and ^13^C MR spectra were processed using MestReNova (Mestrelab Research). Processing entailed automated 0th order phase correction, followed by manual 0th and 1st order fine-tuning, and automated baseline correction. Non-overlapping peaks in both types of spectra were manually integrated whereas the overlapping peaks of pyruvate and acetone in the ^1^H spectrum were resolved by means of peak fitting.

The ^13^C spectra acquired in the *in vitro* enzyme kinetics experiment were stacked and processed similarly. The pyruvate and lactate peaks were clearly non-overlapping therefore manual integration was performed. The resulting pyruvate and lactate time courses were fitted with a Michaelis-Menten type kinetic model using custom python scripts and the apparent first order reaction rates of pyruvate to lactate conversion (k_PL_) were computed from the Michaelis-Menten extracted parameters..

Processing and quantification for all *in vivo* datasets were performed using an in-house tool (NVision), which integrates spatial localization, peak fitting, and metabolite mapping functions. The spectral peaks for pyruvate, lactate, and bicarbonate were integrated and normalized to the pyruvate peak height or area at timepoint zero. In dynamic acquisitions, time courses of metabolic conversion were evaluated by fitting a first-order, two-pool, bi-directional exchange model to the metabolite signal evolution with a smoothed Heaviside input function. For CSI data, spectral maps were reconstructed using a density-weighted Fourier transform and aligned with ^1^H anatomical references. All analyzed data were plotted using GraphPad Prism.

## Supplementary Material

Supplementary Files

This is a list of supplementary files associated with this preprint. Click to download.

- SupplementaryFigures.docx

## Data Availability

Data will be made available upon request.

## Code availability

Code will be made available upon request.
